# Items

**Published:** 1888-02

**Authors:** 


### ITEMS.

Fever-Germs in Drinking Water.—About the 1st of August last, a stranger came to Iron Mountain affected with typhoid fever and died there. No attempts were made to disinfect the surroundings, and from this the fever spread until there have been 350 cases of typhoid fever and thirty-five deaths there. Dr. Vaughan, of the University of Michigan, inoculated sterilized meat preparations and sterilized milk with the Iron Mountain well water, and kept the preparation at the temperature of the body for seven days; a germ developed which was inoculated into some of the lower animals, which became sick with typhoid fever.

It was thus proved that the typhoid fever at Iron Mountain was produced by the use of impure water. Dr. Vaughan says that freezing does not destroy the germ. The ice which the Iron Mountain people used came from a very suspicious source, and no doubt contained germs of typhoid fever. This is the first time that the plan mentioned has been employed to produce the disease in the lower animals from the cause-germ obtained directly from drinking water.

---

Jaborandi as a Galactophore.—A Liege contemporary has called attention to the peculiar influence of jaborandi on the lacteal function. So marked is this said to be that spurious specimens of jaborandi are reported to have been detected from the absence of this property. At the same time its effect is only produced to advantage in a certain class of cases, the precise indications for its use not having been as yet ascertained. In the case of a strong woman, weighing over fifteen stone, whose milk secretion had ceased since more than a fortnight, ten drops of the fluid extract of jaborandi, given every four hours, secured an immediate return of the secretion. Things went on well for a time, but in the course of a few weeks the patient became subject to hallucinations and fits of violent excitement. The jaborandi was suppressed and the symptoms disappeared—and the milk with it.—*Exchange.*

---

It is not generally known that the Emperor of Brazil is suffering from a somewhat advanced stage of diabetes. This makes his extraordinary activity of mind and body all the more remarkable. It is impossible to withhold a tribute of admiration for the indomitable "pluck" of a man who, while suffering from a complaint of the most depressing nature, continues not only to discharge the duties of his high office with unbated vigor, but takes so keen and so comprehensive an interest in all things connected with the progress of knowledge and the furtherance of human happiness.—*Exchange.*

---

Anti-Pyrin is coming more and more into favor, and will soon supercede quinine. Its manifold properties are being discovered daily. M. Chouppe mentioned before the Société de Biologie a case of uterine colic treated successfully by the drug. The case was one of uterine myoma, for which ergot was given in large quantities, causing extremely painful contractions. Thirty grains of anti-pyrin, by the rectum, caused all pain to cease in twenty minutes, although the contractions persisted with the same strength.—*Exchange.*

---

Dr. Manson, an English physician, has been summoned from Hong-Kong to take medical charge of the young Emperor of China, this being the first occasion on which a foreign doctor has ever attended a member of the imperial family.—*Ex.*

---

Professor Grawitz states that *trichina spiralis* have been found in as many as one-third of the cases of so-called muscular rheumatism which have been examined post mortem.—*Ex.*

